# QuickStats

**Published:** 2014-09-19

**Authors:** FROM THE NATIONAL CENTER FOR HEALTH STATISTICS

**Figure:**
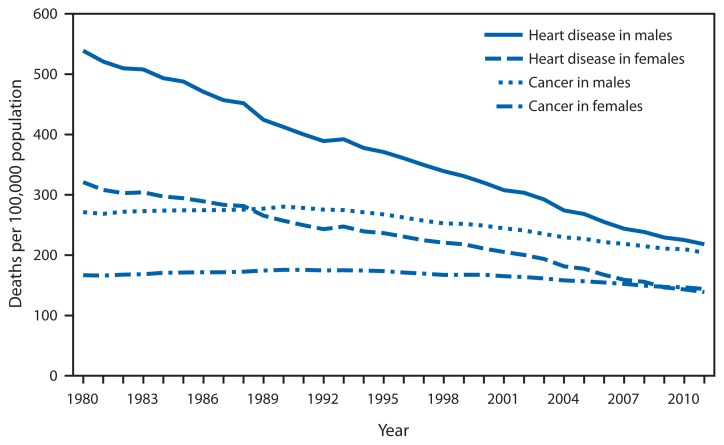
Age-Adjusted Death Rates* for Heart Disease and Cancer,^†^ by Sex — United States, 1980–2011 * Per 100,000 population. ^†^ As the underlying cause of death, heart disease is coded as 390–398, 402, and 404–429 for the period 1980–1998, and I00–I09, I11, I13, and I20–I51 for 1999–2011. As the underlying cause of death, cancer is coded as 140–208 for the period 1980–1998 and C00–C97 for 1999–2011, based on the *International Classification of Diseases, Ninth and Tenth Revisions*.

During 1980–2011, age-adjusted death rates for heart disease in males and females decreased steadily. The rate decreased 59.5% for males and 56.8% for females. In contrast, the rate from cancer first increased 3.4% for males and 5.3% for females during 1980–1990 and then decreased 27.2% for males and 18.0% for females by 2011. For females, the rates for cancer (147.4 per 100,000 population) surpassed the rates for heart disease (146.6) in 2009. The death rate for heart disease in males remained slightly higher (218.1) than the death rate for cancer (204.0) in 2011.

**Source:** National Vital Statistics System. Mortality public use data files, 1980–2011. Available at http://www.cdc.gov/nchs/data_access/vitalstatsonline.htm.

**Reported by:** Jiaquan Xu, MD, jax4@cdc.gov, 301-458-4086.

